# Modeling and Dynamic Parameterized Predictive Control of Dissolved Oxygen in Dual−Tank Bioreactor Systems

**DOI:** 10.3390/bioengineering12070690

**Published:** 2025-06-24

**Authors:** Muhang Li, Ran Tang, Yifei Li, Junning Cui

**Affiliations:** 1 Center of Ultra−Precision Optoelectronic Instrument Engineering, Harbin Institute of Technology, Harbin 150080, China; muhangli218@163.com (M.L.); rantang@hit.edu.cn (R.T.); 2 Key Lab of Ultra−Precision Intelligent Instrumentation, Harbin Institute of Technology, Ministry of Industry and Information Technology, Harbin 150080, China; 3 School of Mathematics, Harbin Institute of Technology, Harbin 150001, China; yifeili@hit.edu.cn

**Keywords:** recirculating culture, dissolved oxygen, dynamic parameterized predictive control, oxygen distribution, bioreactor optimization

## Abstract

Uneven distribution and delayed system response of dissolved oxygen (DO) in dual−tank recirculating bioreactor systems pose significant challenges for oxygen supply. To address these issues, a dynamic parameterized predictive control (DPPC) approach is proposed and validated through simulation and bench−scale experiments. This method is underpinned by a mathematical model that integrates mass transfer kinetics and chemical thermodynamic principles, accurately capturing oxygen dissolution and transfer within a recirculating environment. By predicting future DO variations and continuously integrating real−time monitoring data, the controller adjusts oxygen injection parameters in real time, rapidly restoring DO levels to target values while minimizing overshoot and latency introduced by system circulation. Experimental results in dual−tank setups show an RMSE below 0.05 and an R^2^ exceeding 0.99, affirming the model’s predictive accuracy under varying oxygen conditions. Compared with conventional feedback control strategies, the proposed method demonstrates improved stability, faster response, and lower overshoot, achieving a 47.8% reduction in ISE and a 41.4% reduction in IAE, thus highlighting its superior tracking accuracy. These findings suggest the DPPC method holds promise as a control framework for future application in oxygen−sensitive culture systems.

## 1. Introduction

In cell−based therapies, an in vitro expansion process is typically required, during which strict regulation of environmental conditions is essential to maintain key cellular functions such as viability, proliferation, and differentiation [1,2,3]. Among environmental factors, dissolved oxygen (DO) plays a crucial role in cell culture systems [4,5]. Fluctuations in DO could induce oxidative stress, affect mitochondrial activity, and compromise key cellular functions [6,7]. In addition, cells are sensitive to shear forces, which could activate mechanotransduction pathways and alter cellular fate decisions [8,9]. Therefore, precise DO regulation and shear−induced stress avoidance have become central challenges in the design of advanced bioreactor systems for cells.

Numerous control strategies have been developed to maintain DO levels within a physiologically relevant range (typically 5–10%), including adjustments in agitation speed, gas flow rates, or oxygen fraction in the gas mixture [10,11,12]. While target DO levels can be effectively maintained by these approaches, excessive shear forces are frequently introduced, which may compromise the viability and functional stability of shear−sensitive cells. To mitigate these adverse shear effects, dual−tank recirculating culture systems have been developed, where the aeration and stirring zone is physically separated from the cell growth zone. Such design can reduce localized shear forces while ensuring gas−liquid mixing efficiency; however, it also brings DO gradients between the inflowing and existing medium, along with time delays in responding to adjustments in stirring speed or gas flow rate. These complications often hinder real−time, uniform DO regulation, underscoring the need for more advanced control strategies in dual−tank configurations.

Feedback control methods, such as PID controllers, are widely utilized to maintain target DO levels by comparing real−time measurements with desired setpoints and adjusting operational parameters accordingly [13]. However, in dual−tank recirculating systems, physical separation and fluid transport dynamics lead to both time delays and uneven DO distribution. Conventional feedback control may fail to capture rapid changes in each zone. Moreover, mechanical adjustments (e.g., changing stirring speed or gas composition) often result in transient responses, during which DO levels temporarily deviate from the desired range. Recent efforts in model predictive control (MPC) have been directed toward addressing these limitations by enabling system inputs to be proactively adjusted based on model predictions of future DO demands [12,14]. For example, L. Simon and M.N. Karim [15] employed autoregressive models to optimize agitation speed and gas flow, and A. Kuprijanov [16] proposed a parameter−adaptive MPC with gain scheduling. However, most existing MPC frameworks have been developed under the assumption of near−uniform conditions (i.e., single−tank scenarios), and the DO gradients and extended response times characteristic of dual−tank recirculating systems are not adequately addressed. Consequently, the predictive accuracy and control effectiveness of such models are often degraded in multi−zone environments, where oxygen demand and recirculation flow rates exhibit nonlinear and time−varying behavior.

To tackle these challenges, a novel dynamic parameterized predictive control (DPPC) method was specifically designed for dual−tank recirculating systems. Section 2 outlines the system setup and a mathematical model with transport delays and multi−zone consumption. Section 3 explains the parameterization process. Section 4 presents DPPC validation through simulations and physical experiments using a dual−tank system, demonstrating reduced overshoot, faster settling, and improved DO uniformity compared to conventional control methods. Section 5 summarizes key findings and future directions for broader cell culture applications.

## 2. Theoretical Model Development

### 2.1. Dual−Tank Recirculating System in Cell Culture

**Figure 1:**
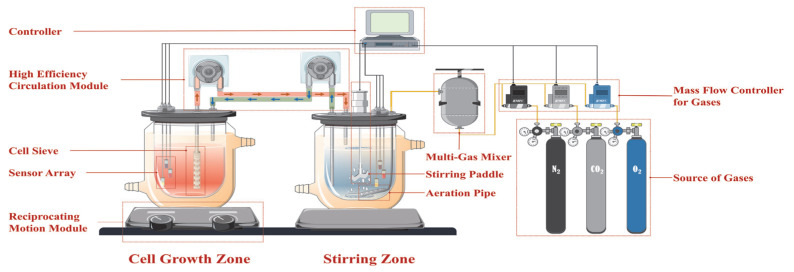
Schematic diagram of the dual−tank recirculating system for cell culture. This diagram illustrates the dual−tank recirculating system designed for cell culture, highlighting its key components and their functions.

To reduce the shear forces caused by stirring and aeration in bioreactors—which may lead to cellular damage and reduced viability—a dual−tank recirculating culture system was adopted. In this system, the cell growth zone is physically separated from the aeration and stirring zone, enabling uniform medium mixing and efficient gas exchange while creating a gentler environment for cell growth (Figure 1).

The dual−tank recirculating system consists of six functional modules designed to optimize key parameters for cell growth. The cell growth zone incorporates a cell sleeve, a sensor array, and a reciprocating motion module, which together support controlled cell agitation and monitoring. In the aeration and stirring zone, a stirring paddle, aeration pipes, and a sensor array promote effective gas–liquid exchange and thorough mixing, ensuring precise oxygen delivery to the culture medium. The high−efficiency circulation module facilitates continuous medium flow between the two zones, preventing gradients in DO and nutrients. Critical environmental parameters, such as temperature, DO, and pH, are continuously monitored and dynamically adjusted by the controller to ensure precise control. Additionally, the gas mass flow controller regulates the proportions of nitrogen (N_2_), oxygen (O_2_), and carbon dioxide (CO_2_) supplied by three cylinders, maintaining a specific gas composition tailored to cellular metabolic demands.

This structural configuration aligns with emerging modular strategies in bioreactor design, as discussed in recent studies. For instance, both Brian Lee [17] and Jin−Sun Lee [18] et al. have proposed the use of dual−stage bioreactor setups in which cell−free medium is diverted to an external oxygenation module or a secondary bioreactor. This approach enables rapid dissolved oxygen enrichment and carbon dioxide removal while avoiding mechanical stress and foam formation that may harm cells. Such designs have been successfully applied in large−scale production systems, demonstrating their feasibility and effectiveness in industrial contexts. These concepts are closely related to the dual−tank configuration adopted in this study. Our design offers a more integrated and controllable solution that not only supports precise and responsive DO regulation under dynamic conditions but also provides a scalable platform adaptable to more complex or industrial bioprocessing scenarios.

To optimize oxygen dynamics within the dual−tank system, a mathematical model was developed to describe changes in DO across the oxygen enrichment and cell growth zones. The developed model predicts DO changes in both zones while dynamically adjusting oxygen input ratios to meet metabolic demands under variable environmental conditions. The mathematical model (Equation (1)) quantifies the time−dependent DO in the stirring zone ($C_{J}$) and the cell growth zone ($C_{Y}$):

(1) $$\left\{\begin{array}{c} \frac{dC_{J}\left(t\right)}{d_{t}}=\frac{V}{V_{L}} [C_{Y}\left(t\right)-C_{J}\left(t\right)]+\lambda_{1}\left(C_{\varphi }^{*}-C_{J}\left(t\right)\right)\\ \frac{dC_{Y}\left(t\right)}{d_{t}}=\frac{V}{V_{L}} [C_{J}\left(t\right)-C_{Y}\left(t\right)]+\lambda_{2}\left(C_{\varphi }^{*}-C_{Y}\left(t\right)\right) \end{array}\right.$$

Here, $C_{J}\left(t\right)$ and $C_{Y}\left(t\right)$ represent the DO (mg/L) in the oxygen enrichment and cell growth zones, respectively. The variable φ represents the oxygen volume fraction, $V_{L}$ denotes the liquid volumes in both zones (L), V indicates the volume of liquid exchanged per minute (L) between the two zones during recirculating culture, and $\lambda_{1}$ and $\lambda_{2}$ are the adjustment coefficients for controlling oxygen input.

### 2.2. Mathematical Modeling of DO in the Cell Growth Zone

Based on the overarching dynamic modeling of DO in the dual−tank recirculating system, the cell growth zone demands particular attention. The dynamic mathematical model was developed to predict and regulate DO fluctuations in real time. Assuming that DO changes exponentially in response to the difference between the saturation level and the current state, the following ordinary differential equation is proposed:

(2) $$\frac{dC_{Y}\left(t\right)}{d_{t}}=k_{L}a\left(C_{\varphi }^{*}-C\left(t\right)\right)$$

In the equation, $\frac{dC\left(t\right)}{d_{t}}$ represents the rate of change of DO in the cell culture medium at time t, measured in mg/L; $k_{L}a$ is the oxygen mass transfer coefficient in the culture medium liquid film, measured in s^−1^; $C_{\varphi }^{*}$ is the saturation DO in the culture medium, measured in mg/L; and $C\left(t\right)$ is the actual DO in the cell culture medium at time *t*, measured in mg/L.

To improve the model’s practical applicability, the model was refined by incorporating expressions for two key parameters directly related to the oxygen volume fraction (φ).

#### 2.2.1. Calculating the Oxygen Transfer Coefficient, $k_{L}a$

In bioreactors, the oxygen mass transfer coefficient, denoted as $k_{L}a$, is a critical parameter that directly affects the efficiency of oxygen transfer to cells. $k_{L}a$ is defined as the product of the total specific surface area available for mass transfer (a) and the liquid mass transfer coefficient ($k_{L}$). The oxygen volume fraction significantly influenced $k_{L}a$, with its relationship to the oxygen transfer rate described by the following equation:

(3) $$k_{L}a=a{\left(\varphi_{O_{2}}\right)}^{m}$$

The coefficient a reflects system properties like oxygen solubility and diffusivity, determining oxygen transfer efficiency, while the exponent m indicates the transfer rate’s sensitivity to oxygen partial pressure changes.

To simplify the complexity of physical processes and isolate key factors influencing mass transfer, the coefficients a and m in Equation (2) are determined using dimensionless analysis. Building on the foundational research of W. Klöckner [19], this study extends the analysis to incorporate the dynamic conditions unique to recirculating culture systems. The refined mathematical expression, presented in Equation (3), integrates these considerations to offer a more precise and accurate prediction of the oxygen volume fraction’s impact on the mass transfer coefficient.

(4) $$\frac{k_{L}a^{2}d_{B}}{g\rho^{2}}=n_{1}{\left(\varphi_{O_{2}}\right)}^{n_{2}}$$

where $\frac{k_{L}a^{2}d_{B}}{g\rho^{2}}$ describes how the O_2_ transfer efficiency is related to hydrodynamic parameters, $d_{B}$ is the inner wall diameter, g is the gravitational accelerationm m/s^2^, and ρ is the liquid density in kg/m^3^. Coefficients n_1_ and n_2_, derived from fitting experimental data, quantified how the φ affected the $k_{L}a$, providing valuable understanding of the system’s oxygen transfer dynamics.

#### 2.2.2. Determining the Saturated DO, $C_{\varphi }^{*}$

To derive a comprehensive equation for the saturated DO ($C_{\varphi }^{*}$) in the culture medium, we consider the key influencing factors, including temperature, pressure, and oxygen volume fraction. Since cell growth typically requires stable temperature and pressure, the oxygen volume fraction ($\varphi_{O_{2}}$) becomes the dominant factor under these conditions. The saturation DO can thus be expressed as follows:

(5) $$C_{\varphi }^{*}=c\varphi_{O_{2}}$$

Here, c is a proportional constant related to the physicochemical properties of the culture medium.

To further refine this relationship, we employ the principle of chemical potential equilibrium between the gas and liquid phases. According to Gibbs free energy principles [20,21], the chemical potential of a molecule in a stable system must be equal across coexisting phases. By combining this equilibrium condition with Dalton’s law of partial pressures, the solubility of oxygen ($S_{O_{2}}$) in the liquid phase is determined as follows:

(6) $$ln\left(S_{O_{2}}\right)=ln\left(P_{O_{2}}\right)-\frac{\mu_{O_{2}}^{l\left(0\right)}\left(T,P\right)-\mu_{O_{2}}^{v\left(0\right)}\left(T,P\right)}{RT}+ln\varphi_{O_{2}}\left(T,P\right)-ln\phi_{O_{2}}\left(T,P\right)$$

Here, $S_{O_{2}}$ represents the solubility of O_2_ in the liquid phase, measured in mol/L. $P_{O_{2}}$ is the partial pressure of O_2_ in the gas phase, measured in Pa. $\mu_{i}^{v\left(0\right)}$ and $\mu_{i}^{l\left(0\right)}$ are the standard chemical potentials of the gas and liquid, respectively, both measured in J/mol [20,21]. *T* represents the environmental temperature (K), P is the pressure (Pa), and R is the Avogadro constant (J/(mol·K)). The fugacity ${(\varphi }_{i})$ and the activity ($\phi_{i})$ coefficients represent how non−ideal behaviors of the substance impact the phases.

In this paper, the fugacity coefficient of O_2_ is calculated using the Helmholtz free energy equation [20,21], while the activity coefficient is obtained through the virial expansion method, which quantifies deviations from ideality in Gibbs free energy [20,22,23]. Combining these coefficients, the saturation DO ($C_{O_{2}}^{*}$) is expressed as follows:

(7) $$C_{O_{2}}^{*}=32\times 10^{3}\times exp\left\{d_{1}ln [\varphi_{O_{2}}\left(P-P_{H_{2}O}\right)]-d_{2}\right\}$$

Here, $d_{1}$ and $d_{2}$ are system−specific constants, $\varphi_{O_{2}}$ is the fugacity coefficient of oxygen, P is the total pressure, and $P_{H_{2}O}$ represents the partial pressure of water vapor. This formulation establishes a reliable framework for predicting the saturation of DO under diverse environmental conditions.

### 2.3. Dynamic Parameterized Predictive Control (DPPC) Method for DO

To precisely regulate DO levels in the cell growth zone, pure oxygen is initially introduced in the stirring zone. This method rapidly restores DO levels by increasing the DO gradient between the two zones, enabling efficient oxygen transfer and minimizing response time. The dynamic DO models established in Equation (1) provide the basis for real−time adjustment of key control parameters, including the oxygen volume fraction (φ) and injection duration ($t_{1}$). These adjustments, guided by model calculations, ensure the target DO in the cell growth zone is reached as quickly as possible.

Once the system stabilizes through initial regulation, finer adjustments are required to maintain DO levels within a precise range and respond to minor fluctuations or disturbances. This is achieved using a feedback control method, which continuously monitors the deviation between target and actual DO levels and adjusts the control input accordingly. The feedback control model, formulated in Equation (8), is expressed as follows:

(8) $$\frac{dY\left(t\right)}{dt}= [aU{\left(t\right)}^{m}] [cU\left(t\right)-Y\left(t\right)]$$

Here, $Y\left(t\right)$ represents the DO at time t, U(t) denotes the control input, and a, m, and c are system−specific parameters.

Due to the nonlinear nature of the model, it was linearized around an equilibrium point, where the system’s derivatives are zero. The linearized form, derived using a first−order Taylor series expansion, calculates the partial derivatives at the equilibrium ${(U}_{0},Y_{0})$ point to represent local changes in the system:

(9) $$\frac{dY\left(t\right)}{dt}\approx \left({\left.\frac{\partial f}{\partial U}\right|}_{U_{0},Y_{0}}\right)\cdot \left(U-U_{0}\right)+\left({\left.\frac{\partial f}{\partial Y}\right|}_{U_{0},Y_{0}}\right)\cdot \left(Y-Y_{0}\right)$$

This linearized model is then converted into a transfer function, enabling a more straightforward analysis of the system’s response. To account for real−world operational conditions, such as sensor and signal transmission delays, the transfer function is augmented with a delay term, resulting in the final form:

(10) $$G_{d}\left(s\right)=G\left(s\right)\cdot e^{-\tau s}=\frac{A}{s-B}\cdot e^{-\tau s}$$

Here, $e^{-\tau s}$ represents the sensor or signal transmission delay, where A = 0.25, B = 0.1, and τ = 10 s, corresponding to the system gain, dynamic pole, and signal transmission delay, respectively. This structure captures both the local dynamics and the inherent delay in DO measurement and control signal propagation.

To ensure a smooth transition to steady−state feedback control, stabilization criteria are defined with a 5% error margin, representing the acceptable deviation between target and actual DO. This threshold reflects the transfer function’s validity near the equilibrium point, where deviations beyond 5% indicate the breakdown of the linear approximation. Maintaining the system within this range ensures stability and minimizes risks of overcorrection. As shown in Table 1, critical DO differences mark the boundaries between stable and unstable states, serving as benchmarks for evaluating system performance under varying conditions.

**Figure 2:**
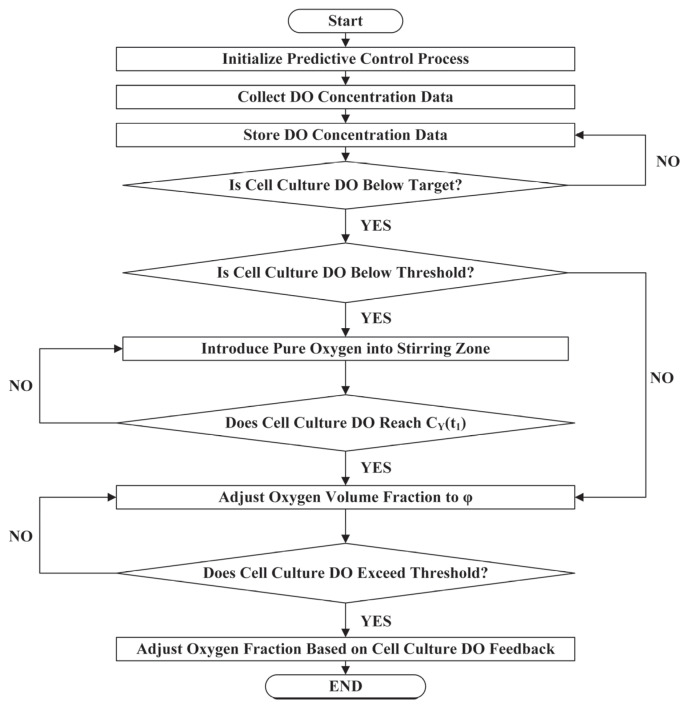
DPPC method regulation flowchart for recirculating cell culture systems.

The DPPC method was developed to adaptively regulate DO levels. To clearly and intuitively present this method, a detailed process flowchart is provided in Figure 2. This flowchart illustrates the dynamic adjustment mechanisms, outlining the step−by−step implementation of the control method.

As part of this framework, a proportional−integral−derivative (PID) controller is employed to achieve fine−tuned regulation of the system. The controller parameters are first initialized using the Ziegler–Nichols ultimate gain method, where the critical gain and oscillation period are identified as $K_{u}$ = 0.1247 and $T_{u}$ = 30 s, respectively. Based on this, the initial parameter estimates are set to $K_{p}$ = 0.06, $K_{i}$ = 0.004, and $K_{d}$ = 0.45. To further refine these parameters for enhanced performance, an offline Simulated Annealing algorithm is applied, with a 1 s sampling interval, explicitly accounting for a 10 s sensor delay. After multiple optimization iterations, the final PID parameters are determined as $K_{p}$ = 0.0015, $K_{i}$ = 0.0005, and $K_{d}$ = 0.01, ensuring robust control performance under dynamic operating conditions. Experimental validation is then conducted to evaluate real−time control effectiveness using these optimized values.

## 3. Experimentally Determined Model Parameterization

**Figure 3:**
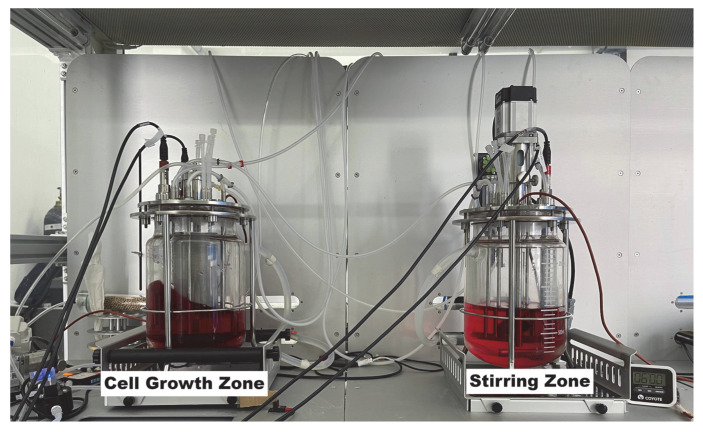
A 3 L dual−tank bioreactor system.

To support model fitting and validate the performance of the DPPC method in regulating DO, a dual−tank bioreactor system was designed and constructed. The two primary functional zones of the system are depicted in Figure 3. Each tank has an effective working volume of 3 L, with the overall system footprint measuring approximately 2.4 m × 0.9 m × 2.2 m (L × W × H).

### 3.1. Determination of the Relationship Between $k_{L}a$ and φ

To investigate the relationship between oxygen volume fractions and dynamic oxygen transfer rates, experiments were conducted under standardized initial conditions. Dissolved oxygen (DO) levels were first reduced to zero by introducing nitrogen (N_2_) into all regions, creating a consistent baseline. Oxygen volume fractions ranging from 5% to 100% were then systematically tested. After establishing these experimental conditions, consistent DO data were collected using sensors and analyzed to calculate the oxygen transfer rate using the following equation:

(11) $$ln\frac{C_{O_{2}}^{*}-C_{O_{2},t_{2}}}{C_{O_{2}}^{*}-C_{O_{2},t_{1}}}=-k_{L}a\times \left(t_{2}-t_{1}\right)$$

Here, $C_{O_{2}}^{*}$ represents the saturation DO (mg/L), $C_{t,O_{2}}$ is the DO at time t (mg/L), and t denotes time.

To simplify the complex interactions among experimental parameters, dimensionless analysis was applied, enabling a focused evaluation of the intrinsic relationships between key variables. Experimental accuracy and consistency were ensured by optimizing key parameters based on system requirements, as summarized in Table 2. These parameters, including shaker speed, medium volume, pH, and temperature, were calibrated to mimic physiological conditions and support effective cell–microcarrier interactions. For example, a medium volume of 1 L provided adequate nutrient supply and waste dilution, while a shaker speed of 150 rpm ensured the complete suspension of cell–microcarrier complexes. Temperature was precisely maintained at 37 °C ± 0.01 °C to replicate the physiological conditions of the umbilical artery [24], fostering cell proliferation and metabolic activity. Additionally, pH stability (7.2 ± 0.1) was achieved by dynamically regulating N_2_ and CO_2_ levels, where CO_2_ adjusted pH through acid–base reactions, and N_2_ indirectly modulated CO_2_.

**Figure 4:**
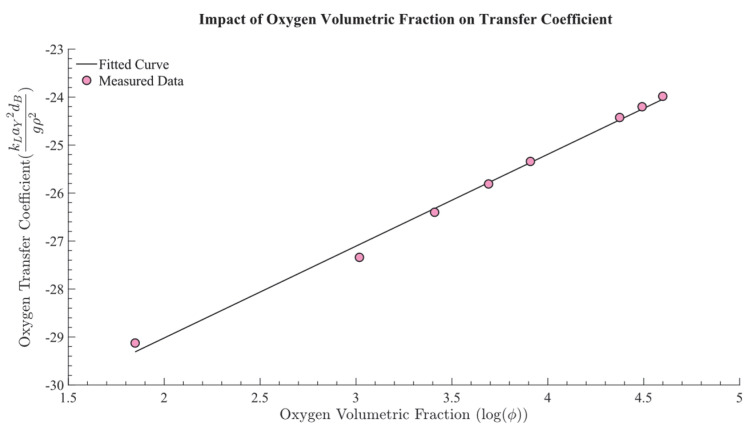
Impact of oxygen volumetric fraction on transfer coefficient. The *x*−axis represents the logarithm of the volumetric oxygen fraction, while the *y*−axis shows the volumetric mass transfer coefficient ($\frac{k_{L}{a_{Y}}^{2}d_{B}}{g\rho^{2}})$.

Using the experimental data, a regression equation was derived to determine the parameters $n_{1}$ and $n_{2}$ in Equation (4), yielding values of $n_{1}$ = 3.23 × 10^−6^ and $n_{2}$ = 3.8278. Figure 4 illustrates the relationship, with the *y*−axis representing the normalized form of the mass transfer coefficient, $\frac{k_{L}{a_{Y}}^{2}d_{B}}{g\rho^{2}}$, showing how $k_{L}a$ increases with higher oxygen volume fractions. This highlights the critical influence of the oxygen volumetric fraction on oxygen transfer efficiency. Additionally, this analysis enabled the determination of the parameters a and m in Equation (3), with a = 2.5302 and m = 1.9139, which are essential for accurately modeling the system’s oxygen transfer behavior.

### 3.2. Determination of the Relationship Between $C_{\varphi }^{*}$ and φ

**Figure 5:**
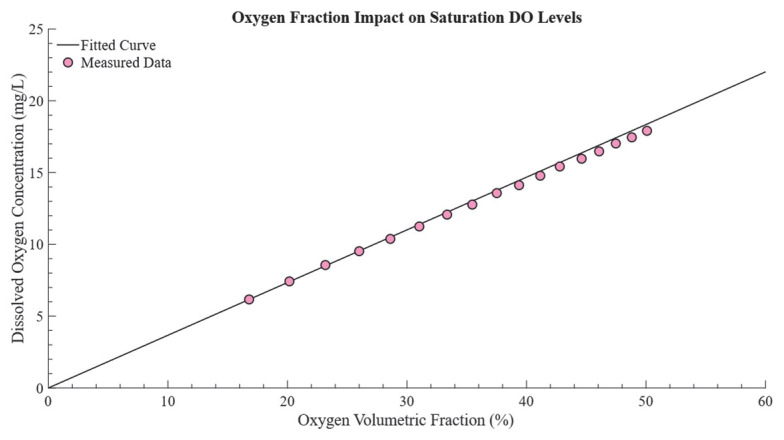
Correlation between oxygen volume fraction and saturated DO under controlled conditions. The *x*−axis shows the oxygen volume fraction, and the *y*−axis shows saturated DO (mg/L). Solid lines represent the fitted curves, while dots denote the experimental measurements, where the dots represent average experimental values, and the solid black line shows the least squares fitted curve.

For each oxygen volume fraction, five independent experiments were performed to measure the corresponding saturated DO. The average values from these experiments were plotted, with the oxygen volume fraction on the *x*−axis and the saturated DO on the *y*−axis, as shown in Figure 5. By fitting the experimental data to a curve, the parameters $d_{1}$ = 0.0022, $d_{2}$ = 4.6525, and c = 36.1053 were determined. These parameters were subsequently applied to Equation (7) to facilitate accurate future calculations of pressure−dependent saturated DO.

### 3.3. Coefficient Determination for the Mathematical Model of the Recirculating Culture System

**Figure 6:**
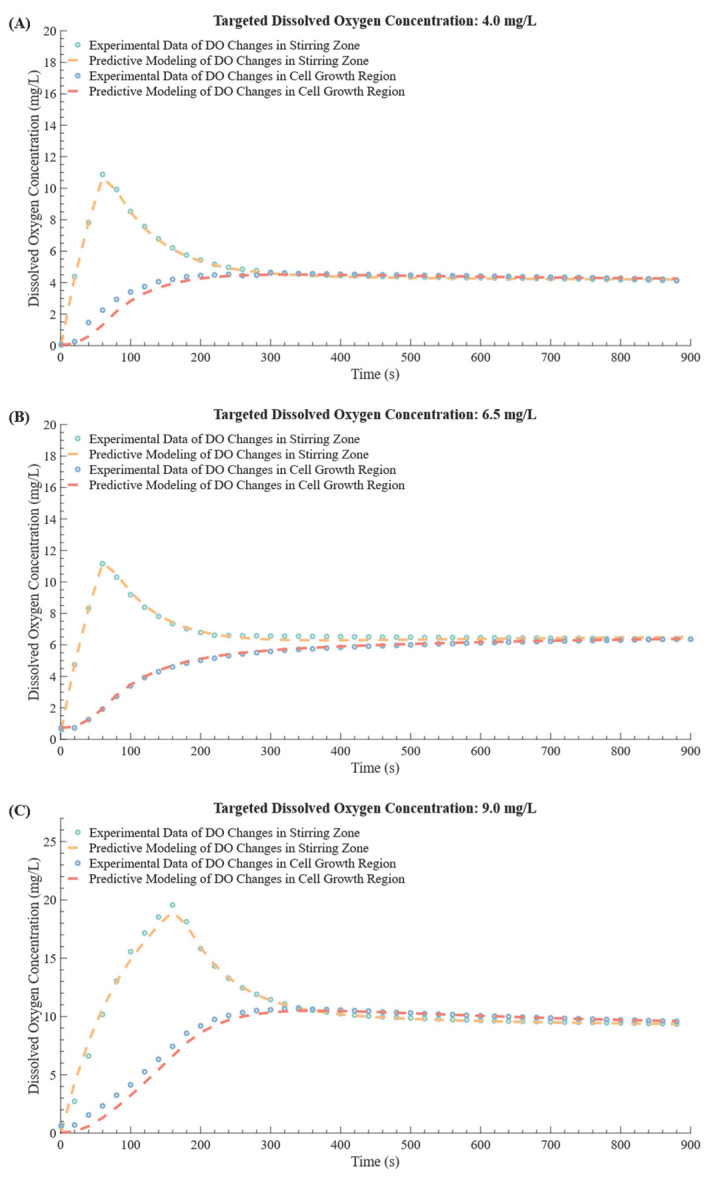
Comparison between the predictive model and experimental data for DO in the recirculating culture system. The figure presents a comparison of the experimental DO data in the aeration and stirring zone and the cell growth zone against the predictive model. (**A**) Target DO of 4.0 mg/L, (**B**) Target DO of 6.5 mg/L, (**C**) Target DO of 9.0 mg/L.

To describe the dynamic changes in DO within the dual−tank recirculating culture system, a mathematical model was developed, as outlined in Section 2.1. During the recirculation process, target DO levels of 4.0 mg/L, 6.5 mg/L, and 9.0 mg/L were set, with oxygen introduced at specific volume fractions calculated using the model to achieve these targets. Sensors monitored DO levels in both regions of the system, and the experimental data were used to fit the dynamic model, leading to the determination of key parameters: $\lambda_{1}$ = 0.0069233 and $\lambda_{2}$ = 0.0004899. The fitting results, illustrated in Figure 6, show strong alignment between model predictions (based on Equation (1)) and experimental data across all targets, demonstrating the model’s ability to capture the system’s dynamic response.

To further validate the model, two performance metrics were applied: Root Mean Square Error (RMSE) and the coefficient of determination (R^2^), as summarized in Table 3. RMSE, which quantifies the average deviation between model predictions and experimental data, was calculated for the three targets, yielding values of 0.0472, 0.0465, and 0.0415, all below the 0.05 threshold. Similarly, R^2^ values, which measure how well the model explains variance in the experimental data, were close to 1, with values of 0.9986, 0.9929, and 0.9959, respectively. These metrics confirm the strong agreement between the model predictions and experimental outcomes, validating its accuracy in simulating DO dynamics in the dual−tank system. Notably, the achieved R^2^ values exceed those reported in other recent studies. For instance, Kuo−Chun Chiu et al. [25] developed a DO prediction model based on a NeuralODE framework and reported an R^2^ of 0.91, while Tianyu Lu et al. [26] proposed a CNN–LSTM hybrid deep learning architecture that achieved an R^2^ of 0.956. Compared to these approaches, the proposed model demonstrates superior predictive performance with higher fitting accuracy.

The identified parameters and validation results significantly enhance the model’s reliability, providing a robust foundation for the DPPC system. In the subsequent sections, the DPPC method’s performance will be evaluated through targeted experimental verification, further demonstrating its practical applicability.

## 4. Control Performance Assessment of DDPC

### 4.1. Simulation−Based Experimental Verification

**Figure 7:**
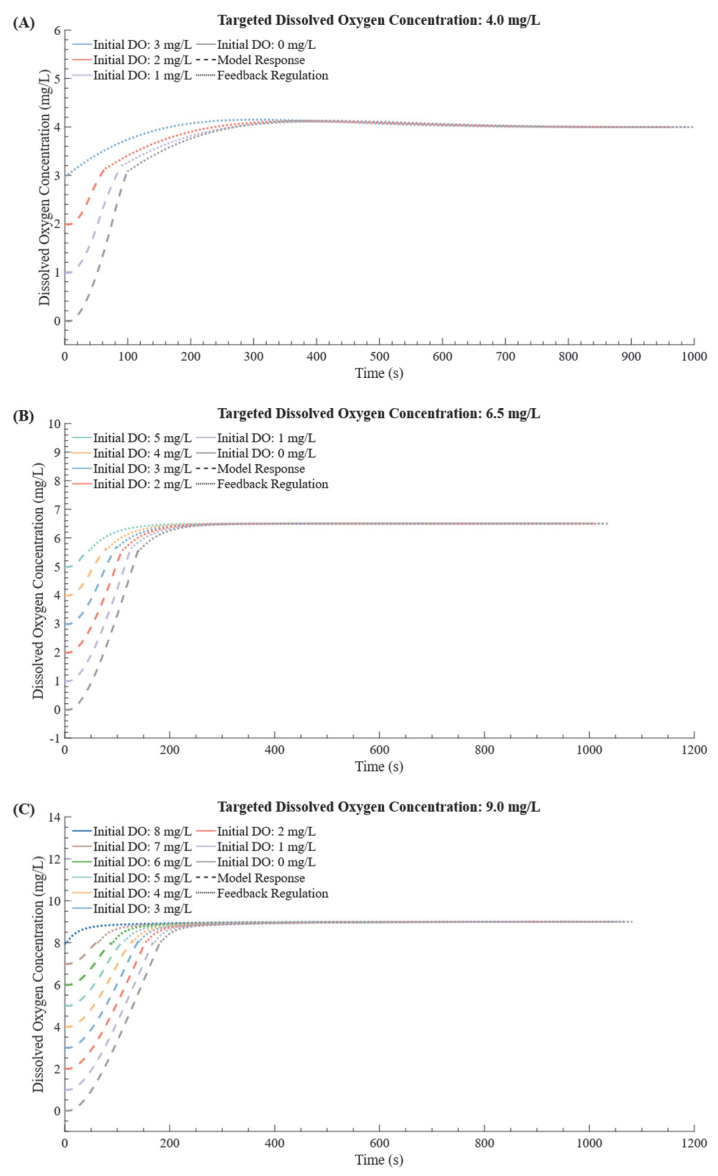
Simulation results of DO control methods for various initial conditions and targets. The DO targets are 4.0 mg/L (**A**), 6.5 mg/L (**B**), and 9.0 mg/L (**C**), as shown in the corresponding subplots. Each graph uses different colored lines to represent various starting DO levels. The dashed lines represent the adaptive component of the DPPC method, while the dotted lines indicate the predictive component.

Simulation experiments were conducted to evaluate the performance of the DPPC model across varying initial and target DO levels. Initial DO values were incrementally set from 0 mg/L to 8 mg/L, covering a range from zero oxygen to near−target conditions, as shown in Figure 7. In the figure, dotted lines represent the feedback control component, which provides precise real−time adjustments, while dashed lines denote the predictive control component, designed to anticipate and manage larger deviations from the target. Together, these components illustrate the dual functionality of the DPPC method. The model consistently achieved convergence to target DO levels, with an average settling time of 376 s and a steady−state error of 0.2 mg/L across all initial conditions. Response times were largely uniform, with minor variations observed for initial DO values close to zero or significantly above the target, reflecting the model’s ability to adapt across a broad range of scenarios. These findings highlight the DPPC model’s robust adaptability, demonstrating its potential for precise control in dynamic biological systems where DO levels fluctuate significantly. Previous studies have primarily explored DO regulation under low initial concentrations in aquaculture settings, whereas this aspect has received limited attention in cell culture applications. For instance, Zhou et al. [27] reported significant overshoot and variable settling times when applying PID−based tuning under similar step changes in DO, especially at low starting concentrations. Compared to that, the DPPC model demonstrated more consistent settling behavior and tighter control margins, confirming its adaptability under challenging starting conditions.

**Figure 8:**
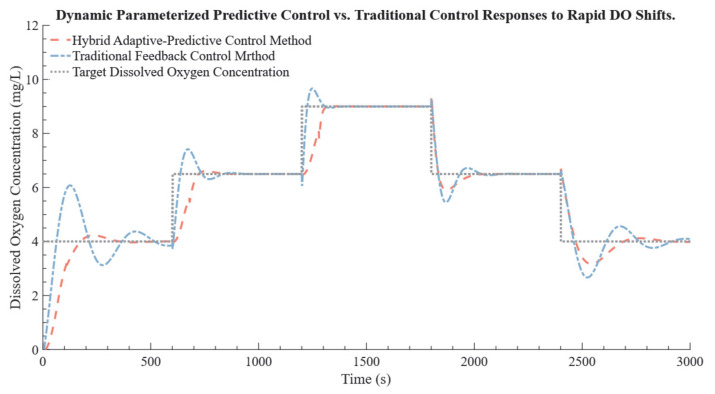
Comparison of DPPC and traditional feedback control responses to rapid DO target shifts. The *x*−axis represents time in seconds (s), indicating the duration over which DO adjustments were made. The *y*−axis represents DO in milligrams per liter (mg/L), showing the level of DO achieved by each control method. In the plot, the red dashed line denotes the response of the DPPC method, the blue dashed line indicates the response of the traditional feedback control method, and the gray dotted line represents the target DO for each interval.

To simulate rapid environmental changes, the target DO is adjusted every 600 s, as shown in Figure 8. In this figure, the gray line represents the target DO levels, while the red and blue lines illustrate the responses of the DPPC method and traditional feedback control, respectively. Table 4 presents a quantitative comparison between the two control methods. Specifically, the DPPC method reduced overshoot by 37.84% to 100% across various peak shifts, with some cases completely eliminating overshoot, indicating a significant improvement in control precision. Additionally, during the 6.5–9.0 mg/L transition, it completely eliminated overshoot, underscoring its improved control precision. The hybrid control consistently reached the target levels more quickly, achieving an average settling time shorter than that of the traditional feedback control. In contrast, traditional feedback control exhibited slower response times with substantial overshoot and oscillations, particularly during large target shifts, such as in the 0–4.0 mg/L transition, where significant oscillations were observed before stabilization. Overall, the DPPC method demonstrated superior accuracy, establishing it as a robust choice for managing rapid DO fluctuations. These findings suggested that the DPPC method was particularly suitable for biological systems requiring precise DO regulation, such as in bioreactors or dynamic cell culture environments where oxygen demand fluctuated rapidly.

To quantitatively assess control performance, two commonly used evaluation metrics were employed: the Integral of Squared Error (ISE) and the Integral of Absolute Error (IAE). ISE captures both the magnitude and duration of deviations, making it particularly sensitive to large transient errors. In contrast, IAE reflects the overall tracking performance over time. In this test, the PID controller yielded an ISE of 4284.10 and an IAE of 2147.12, whereas the DPPC method achieved significantly lower values, with an ISE of 2233.98 and an IAE of 1257.82. These results correspond to a 47.84% reduction in ISE and a 41.41% reduction in IAE, demonstrating that the DPPC method not only improves response speed but also achieves higher accuracy and smoother control during dynamic DO target shifts.

This simulation focused on evaluating the model’s stability in maintaining DO levels amid metabolic fluctuations induced by oxygen consumption during cell culture. The generalized logistic equation proposed by L. Mancuso [28] was employed to model stem cell proliferation, where a random function to simulate dynamic oxygen consumption fluctuations is applied, incorporating stem cell proliferation parameters along with a random function to introduce dynamic oxygen consumption fluctuations. To link cell proliferation with oxygen dynamics, the Monod−based model from E. Bartolini [29] Equation (12) was integrated into the DO calculations. This equation incorporates key factors such as the oxygen transfer rate (OTR) and oxygen uptake rate (OUR), allowing the simulation of real−time DO adjustments:

(12) $$\frac{dC_{Y}\left(t\right)}{d_{t}}=OTR\left(t\right)-OUR\left(t\right)=k_{L}a\left(C_{\varphi }^{*}-C\left(t\right)\right)-\frac{OUR_{max}\cdot C\left(t\right)}{K_{s}+C\left(t\right)+K_{N}\cdot N}$$

Here OUR represents the oxygen uptake rate per unit time for the cells, $OUR_{max}$ denotes the maximum oxygen uptake rate, K is the Monod constant influencing substrate availability, $K_{N}$ indicates the impact of cell number on oxygen uptake, and N is the total cell count.

**Figure 9:**
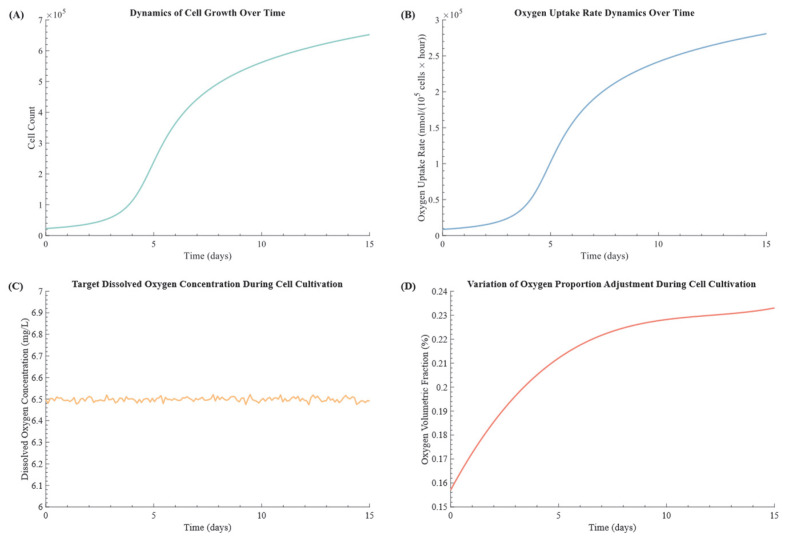
The 15−day simulation of dynamic control in cell culture. (**A**) Stem cell growth curve: illustrated the steady increase in cell numbers over time. (**B**) Oxygen consumption rate: reflected the rise in total oxygen uptake as cells proliferated. (**C**) DO control: demonstrated precise tracking of DO levels within the target range. (**D**) Oxygen ratio adjustment: highlighted the control method’s flexibility and responsiveness in dynamically adjusting oxygen ratios based on cellular oxygen demand.

The simulation results, shown in Figure 9, highlight the system’s performance over a 15−day culture period. Figure 9A displays the stem cell growth curve, following a characteristic S−shaped trajectory as cell proliferation accelerates and saturates. This proliferation increased oxygen demand, reflected in Figure 9B as a corresponding rise in total oxygen uptake rate. To meet the growing respiratory needs, the system maintained a stable DO level at 6.5 mg/L (Figure 9C), with fluctuations tightly controlled within 6.5 ± 0.05 mg/L. The fluctuation range of 0.05741 mg/L and a stability factor of 0.00991 demonstrate the model’s exceptional precision and reliability. As cell numbers and oxygen consumption rose, the DPPC method dynamically adjusted the oxygen volume fraction in the input gas, as shown in Figure 9D. This responsiveness ensured that oxygen availability remained aligned with cellular needs, supporting optimal growth and function throughout the culture period. These results underscore the robustness and adaptability of the DPPC method, confirming its suitability for dynamic biological systems requiring precise oxygen regulation. In particular, the proposed method maintained DO levels with a high degree of precision (±0.05 mg/L), outperforming recent adaptive disturbance−rejection control strategies, such as the one reported by Yao et al. [30], which achieved a control accuracy of ±0.095 mg/L. This higher level of precision, combined with the system’s modular architecture, provides a more scalable and flexible solution for long−term cell culture applications.

### 4.2. Experimentally−Based System Verification

**Figure 10:**
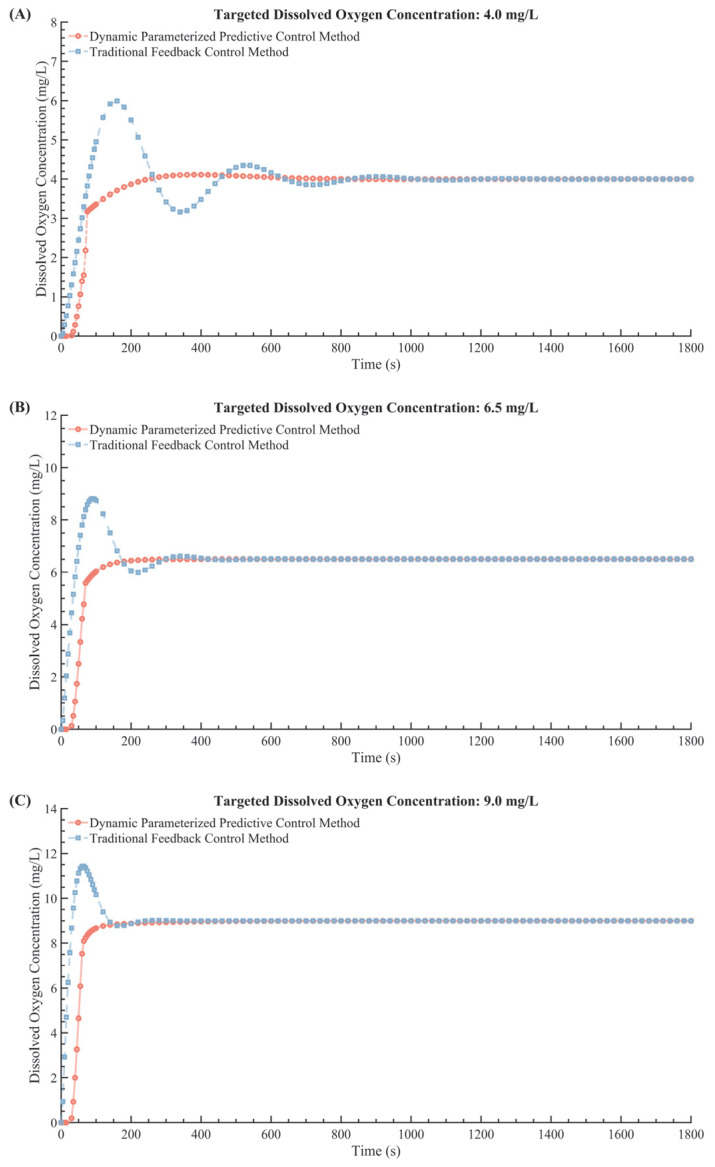
Comparative responses of control methods at target DO levels. Panels (**A**–**C**) correspond to DO targets of 4.0 mg/L, 6.5 mg/L, and 9.0 mg/L, respectively. The red curve represents the DPPC, while the blue curve represents traditional feedback control.

Following successful validation in simulation experiments, the DPPC method was further tested through multi−target experiments in a real dual−tank recirculating culture system. These experiments aimed to evaluate its performance under three specific DO targets—low (4.0 mg/L), medium (6.5 mg/L), and high (9.0 mg/L)—to meet the diverse requirements of cell cultures [31,32,33]. The results demonstrated that the hybrid method consistently outperformed the traditional control method in both stabilization speed and overshoot reduction. For the 4.0 mg/L target, the hybrid method achieved stability in 139 s with only 2.8% overshoot, while the traditional method required 412 s and exhibited a significant 49.94% overshoot. At the medium target of 6.5 mg/L, the hybrid method stabilized within 108 s with zero overshoot, compared to 243 s and 35.7% overshoot for the traditional method. Similarly, for the highest target of 9.0 mg/L, the hybrid method reached stability in 92 s without overshoot, while the traditional method needed 198 s and showed 27.15% overshoot. These results, visually corroborated in Table 5 and Figure 10, highlight the superior performance of the hybrid control method, which demonstrated faster stabilization, reduced oscillations, and minimal overshoot across all tested conditions. The marked improvements in settling time and accuracy underline the method’s suitability for bioprocessing applications that demand precise and stable DO regulation, particularly in sensitive cell culture environments where rapid adaptability and control precision are critical for maintaining optimal cell growth and differentiation.

## 5. Conclusions

This paper developed and validated the DPPC method for regulating DO in a dual−tank recirculating culture system specifically designed for cell applications. The proposed method demonstrated significant advantages over traditional feedback control by achieving enhanced stability, faster response times, and minimized overshoot, making it highly effective in managing DO fluctuations in dynamic cell culture environments. The findings underscored the hybrid control method’s adaptability and precision, aligning oxygen availability with cellular demands and maintaining optimal conditions for cell growth. This approach offers a robust solution for bioprocessing applications requiring stringent oxygen regulation, particularly in sensitive biological systems where fluctuations in oxygen levels can impact cellular health and productivity. Overall, the DPPC method provides a promising tool for improving control accuracy in oxygen−sensitive environments.

## Figures and Tables

**Table 1:** Critical DO and error margins for stability assessment.

Target DO Range (mg/L)	Critical DO Difference (mg/L)	Regional Error (Maximum)
0–5.42	0.60	4.58%
5.42–12.64	1.00	4.86%
12.64–18.05	1.50	4.07%
18.05–25.27	2.00	4.71%
25.27–32.49	2.50	4.32%
32.49–36.11	3.00	4.80%

**Table 2:** Experimental parameters optimized for replicating physiological conditions and facilitating oxygen transfer analysis.

Variable	Description	Setting or Range	Unit
Shaker Speed	Ensures complete suspension of cell–microcarrier complexes.	150	rpm
Culture Medium Volume	Supports sufficient nutrient supply and waste dilution.	1	Liters
Simulated Arterial Pressure	Mimics arterial pressure in human umbilical cord.	80 ± 5	mmHg
Nitrogen Volume Fraction	Adjusted to maintain optimal pH in the culture area.	Adjusted as needed	Percentage
Carbon Dioxide Volume Fraction	Adjusted to maintain optimal pH in the culture area.	Adjusted as needed	Percentage
Oxygen Volume Fraction	Studies the relationship between oxygen transfer rates and volume fraction.	0%, 10%, 20%, 30%, 40%, 50%	Percentage
pH	Maintains cellular activity and experimental accuracy.	7.2 ± 0.1	pH Units
Temperature	Consistency with cell growth experiments.	37 ± 0.1	°C
Reactor Inner Wall Diameter	Specifies the physical dimension of the bioreactor.	0.128	Meters
Gravity Acceleration	Affects the sedimentation and distribution of cells and particles.	9.0866	m/s^2^
Liquid Density	Impacts fluid dynamics and cell settling.	1250	kg/m^3^

**Table 3:** Evaluation of predictive models for DO in recirculating cultivation.

DO Target (mg/L)	RMSE	R_2_
4.0	0.0472	0.9986
6.5	0.0465	0.9929
9.0	0.0415	0.9959

**Table 4:** Performance comparison of control models across rapidly changing targets.

DO Change Program (mg/L)	Dynamic Parameterized Predictive Control Method Overshoot	Traditional Feedback Control Method Overshoot	Percentage Reduction
0.0–4.0	5.62%	51.73%	89.13%
4.0–6.5	2.80%	21.79%	87.15%
6.5–9.0	0.00%	7.23%	100%
9.0–6.5	0.72%	9.14%	92.12%
6.5–4.0	20.73%	33.35%	37.84%

**Table 5:** Performance comparison of the control method at various DO targets.

Model	Targeted DO (mg/L)	Settling Time (s)	Overshoot
Dynamic Parameterized Predictive Control Method	4.0	139	2.80%
	6.5	108	0.00%
	9.0	92	0.00%
Traditional Feedback Control Method	4.0	412	49.94%
	6.5	243	35.7%
	9.0	198	27.15%

## Data Availability

The original contributions presented in this study are included in the article. Further inquiries can be directed to the corresponding author.
